# Validation of a next-generation sequencing (NGS) panel to improve the diagnosis of X-linked hypophosphataemia (XLH) and other genetic disorders of renal phosphate wasting

**DOI:** 10.1530/EJE-20-0275

**Published:** 2020-08-14

**Authors:** Susanne Thiele, Ralf Werner, Annika Stubbe, Olaf Hiort, Wolfgang Hoeppner

**Affiliations:** 1 Division of Paediatric Endocrinology and Diabetes, Department of Pediatrics, University of Lübeck, Lübeck, Germany; 2 Institute for Molecular Medicine, University of Lübeck, Lübeck, Germany; 3 Labor Dr. Heidrich und Kollegen MVZ GmbH, Hamburg, Germany; 4 Bioglobe GmbH, Hamburg, Germany

## Abstract

**Background:**

Hypophosphataemic rickets (HR) comprise a clinically and genetically heterogeneous group of conditions, defined by renal-tubular phosphate wasting and consecutive loss of bone mineralisation. X-linked hypophosphataemia (XLH) is the most common form, caused by inactivating dominant mutations in *PHEX*, a gene encompassing 22 exons located at Xp22.1. XLH is treatable by anti-Fibroblast Growth Factor 23 antibody, while for other forms of HR such as therapy may not be indicated. Therefore, a genetic differentiation of HR is recommended.

**Objective:**

To develop and validate a next-generation sequencing panel for HR with special focus on *PHEX*.

**Design and methods:**

We designed an AmpliSeq gene panel for the IonTorrent PGM next-generation platform for *PHEX* and ten other HR-related genes. For validation of *PHEX* sequencing 50 DNA-samples from XLH-patients, in whom 42 different mutations in *PHEX* and 1 structural variation** have been proven before, were blinded, anonymised and investigated with the NGS panel. In addition, we analyzed one known homozygous *DMP1* mutation and two samples of HR-patients, where no pathogenic *PHEX* mutation had been detected by conventional sequencing.

**Results:**

The panel detected all 42 pathogenic missense/nonsense/splice-site/indel *PHEX*-mutations and in one the known homozygous *DMP1* mutation. In the remaining two patients, we revealed a somatic mosaicism of a *PHEX* mutation in one; as well as two variations in *DMP1* and a very rare compound heterozygous variation in *ENPP1* in the second patient.

**Conclusions:**

This developed NGS panel is a reliable tool with high sensitivity and specificity for the diagnosis of XLH and related forms of HR.

## Introduction

X-linked hypophosphataemia (XLH) (OMIM#307800) is the most common genetic disorder of phosphate homeostasis characterized by renal phosphate wasting and hypophosphataemia. It affects about one in 20 000 individuals (1) and follows an X-chromosomal dominant inheritance pattern.

Children affected by XLH present with a broad phenotypic spectrum ranging from isolated hypophosphatemia with few clinical signs up to severe symptoms, such as rickets with extreme lower limb deformities, distinct tooth problems (such as dental abscesses), and a disproportionate short stature (reviewed in (2)). In adulthood, further symptoms may occur, such as osteomalacia, arthrosis, pseudo fractures, and diminished final height (3). Further clinical signs are hearing difficulties, enthesopathy, and muscular dysfunction. As the disorder is more under focus during the last few years, more clinical signs have been associated with XLH, such as Arnold–Chiari malformation and other craniofacial abnormalities. Furthermore, bone pain is a very pronounced sign in XLH, leading to an impairment of the quality of life in affected children and adults (reviewed in (2)).

XLH is caused by mutations in the *PHEX* gene encoding the cleavage enzyme phosphate-regulating neutral endopeptidase (PHEX) located on the X chromosome. Today, more than 588 mutations have been reported, spread all over the 22 exons of the *PHEX* gene (http://www.hgmd.cf.ac.uk/). This includes point mutations, deletions, insertions, as well as intronic variations, presumably altering PHEX function. Although the pathophysiology of XLH is not fully understood, the inactivation of the PHEX protein (expressed predominantly in osteoblasts) leads to an increase of fibroblasts growth factor 23 (FGF23) levels. High FGF23 levels cause urinary phosphate wasting by down-regulating the renal sodium phosphate transporters 2a and 2c (NaPi2a and NaPi2c, respectively) and reducing transformation of 25-OH-Vitamin D_3_ to the active vitamin D form 1,25-OH_2_-Vitamin D_3_ (4, 5, 6) leading to abnormal low levels of 1,25-OH_2_-Vitamin D_3_ despite of hypophosphatemia. Therefore, laboratory hallmarks in XLH are hypophosphataemia, reduced renal-tubular phosphate reabsorption, and inappropriately low to normal 1,25 (OH)_2_-vitamin D_3_ levels. In addition, elevated alkaline phosphatase is seen as a marker of higher bone turnover related to rickets.

There is a high overlap between XLH and other forms of HR both in clinical as well as in laboratory findings. Some of those components that have been associated with elevated FGF23 expression or decreased degradation of FGF23 include (amongst others) FAM20C (family with sequence similarity 20 member C) (7), furthermore, ENPP1 (ectonucleotide pyrophosphatase/phosphodiesterase) encoded by the *ENPP1* gene (8), and DMP1 (dentin matrix acidic phosphoprotein 1) encoded by the *DMP1* gene (9, 10). Consequently, inactivating mutations in these genes also lead to an elevation of serum FGF23 levels and to disorders with a similar phenotype to XLH. A further known pathomechanism is caused by increased FGF23 levels due mutations in *FGF23* itself, which affect the cleavage site for degradation. This condition follows an autosomal-dominant (AD) inheritance and the phenotype seems to be milder (11). In contrast to XLH, ADHR shows incomplete penetrance, variable age at onset, and vanishing of the phosphate-wasting defect in rare cases (11, 12, 13). Table 1 summarizes different forms of HR with their biochemical characteristics in comparison to nutritional rickets.

**Table 1:** Different types of hypophosphataemic rickets (HR), their abbreviations, genetic origin, and biochemical features.

Name	Abbreviation	Gene	FGF23	Serum-P	Serum-Ca	Urine-P	Urine-Ca	25OHD3	1,25OHD3	PTH	AP
X-linked hypophosphataemia	XLHR/XLH	*PHEX*	↑	↓	–	↑	↓	–	↓*	–	↑
Autosomal dominant hypophosphataemic rickets	ADHR	*FGF23*	↑	↓	–	↑	↓	–	↓*	–	↑
Autosomal recessive hypophosphataemic rickets 1	ARHR1	*DMP1*	↑	↓	–	↑	↓	–	↓*	–	↑
Autosomal recessive hypo-phosphataemic rickets 2	ARHR2	*ENPP1*	↑	↓	–	↑	↓	–	↓*	–	↑
Hereditary hypophosphataemic rickets with hypercalciuria (HHRH)	HHRH	*SLC34A3*	↓	↓	–	↑	↑	–	↑	↓	↑
Nutritional rickets	NR	–	–	↓	–	–	↓	↓↓	–	↑↑	↑↑↑

–, in general in the normal range; ↑, elevated; ↓, decreased; ↓* inadequate in the lower normal range.

Conventional therapy of HR includes oral phosphate supplementation and, in forms with FGF23-mediated hypophosphatemia, calcitriol; however, this therapy further stimulates FGF23 excretion, enforcing the renal phosphate wasting (6). Recently, a novel therapy with an anti-FGF23 antibody has been approved and current results demonstrate an enormous impact on medical outcome for patients with XLH in children (14, 15, 16) and adults (17). The novel therapy with Burosumab has only been approved for XLH, and it is currently unknown if patients with other forms of HR with FGF23 elevation might profit or not from this therapy. Some forms of HR, for example, caused by *ENPP1* mutation, may even have unfavorable effects from Burosumab such as hypercalcemia and calcification, although this is also currently unknown.

In XLH, early diagnosis followed by an immediate treatment has a strong impact on the patient’s long-term outcome (18). However, the diagnosis of this rare condition is often delayed. The first clinical signs are often mild and occur when a toddler starts standing alone and walking, leading to bowing of the lower limbs, which can also be the first sign of rickets due to vitamin D deficiency. Even the biochemical signs are not always straight forward and may altogether not differentiate between XLH and other forms of HR (19). Therefore, the molecular genetic confirmation of the clinically and biochemically based diagnosis of XLH and differentiation from other forms of HR has been recommended by many specialists of this disorder (2). Until today, the gold standard for the search of mutations in the *PHEX* gene has been Sanger sequencing of all 22 exons, including the exon/intron boundaries for detecting splice site mutations, followed by multiplex ligation-mediated probe amplification analysis (MLPA). By this approach, the diagnosis is relatively expensive and time-consuming and cannot detect other forms of HR. If several patients were to be analyzed in one approach and several genes of one panel were to be examined for some of the patients, we were able to determine a total time and cost-saving from 10 samples onward by NGS analysis.

For these reasons, we developed a next-generation sequencing (NGS) panel comprising not only all exons and the intron boundaries of the *PHEX* gene but also ten other genes, in which mutations are known to cause renal phosphate wasting disorders to ensure the distinct differential diagnosis of XLH.

## Methods

The ethical committee of the University of Lübeck approved this part of the study in January 2004 (04-020) and confirmed the ethical correctness for developing of a NGS panel with the same samples in October 2018 (18-271).

### DNA samples with known ***PHEX***-mutations

For validation of the gene panel, we involved DNA samples from 50 XLH patients with a confirmed clinical, biochemical, and molecular genetic diagnosis representing 42 different *PHEX* mutations. The mutations cover a broad spectrum of short deletions, missense, nonsense and splice site mutations covering most of the exons of the *PHEX* gene, as well as one large duplication of exon 12. The samples were anonymized and blinded before inclusion into the panel investigation. DNA had been prepared from EDTA-blood with Qiagen blood kit (Qiagen).

After validation of the gene panel for *PHEX,* we analyzed three additional samples in which HR had been diagnosed by clinical and biochemical signs in the patients, but without a detectable mutation in *PHEX* analyzed by Sanger sequencing. One sample has a known homozygous variant in the *DMP-1* gene. All 53 samples were anonymized with continuous numbers, only harboring the information of the sex chromosomes differentiation between male and female samples.

### Establishing the IonTorrent AmpliSeq gene panel

We utilized the IonTorrent PGM next-generation platform (Thermo Fisher Scientific) in our setting for molecular diagnosis of XLH. The NGS was performed according to the standard protocol recommended by the system supplier.

The gene panel was designed using the AmpliSeq Designer online tool from Thermo Fisher Scientific (https://www.ampliseq.com/). Technical characteristics of the gene panel are shown in Table 2 and Supplementary Table 1 (see section on supplementary materials given at the end of this article). Patient DNA was amplified by multiplex PCR. We included 11 genes in our gene panel of HR-related disorders – *PHEX, FGF23, DMP1, ENPP1, SLC34A3, CLCN5, SLC34A1, SLC9A3R1, FAM20C, FGFR1* and *KL* – which are involved in phosphate metabolism or are known to cause different types of HR. Sequence analysis was carried out with the software module SeqNext (SeqPilot™, JSI, Ettenheim, Germany). Small gaps in the designed panel, mainly due to large homopolymer stretches, were complemented by Sanger sequencing (Table 2 and Supplementary Tables 2, 3).

**Table 2:** Technical characteristics of the next-generation sequencing (NGS) panel for X-linked hypophosphataemia.

	Ref Seq	Number of exons	Number of homopolymers	Characteristics
Genes				
*PHEX*	NM_000444.5	22	2	
*CLCN5*	NM_000084.,4	11	0	
*DMP1*	NM_004407.3	5	1	
*ENPP1*	NM_006208.2	25	6	
*FAM20C*	NM_020223.3	10	4	
*FGF23*	NM_020638.2	3	0	
*FGFR1*	NM_023110.2	17	0	
*KL*	NM_004795.3	5	4	
*SLC34A1*	NM_003052.4	12	3	
*SLC34A3*	NM_080877.2	12	1	
*SLC9A3R1*	NM_004252.4	6	1	
NGS Panel details				
Number of genes				11
Panel size				45.98 kb
Primer Pools				2
Total number of exons				128
Total number of amplicons				245
Amplicon lengths				125–275 bp
Coverage				99.88%

### Multiplex ligation-mediated probe amplification (MLPA)

Large deletions or duplications encompassing one or more exons of *PHEX* can be analyzed by MLPA. The MLPA reaction was performed according to the standard protocol recommended by the system supplier (Salsa MLPA probemix P223, MRC-Holland, Amsterdam, The Netherlands) (20). The evaluation was carried out with GeneMarker (SoftGenetices, State College, USA).

### iPLEX and MALDI-TOF MS

The mosaic mutation was confirmed by using iPLEX and MALDI-TOF MS (Agena Bioscience, Hamburg, Germany). The iPLEX reaction was performed according to the standard protocol recommended by the system supplier (21). The homogeneous MassEXTEND (hME) and iPLEX process reley on a small volume PCR amplifying the target regions including the SNP position in a multiplex fashion. The basic principle of hME and iPLEX reaction is identical. Both methods use a third, so-called MassEXTEND primer, which anneals directly adjacent to the SNP position. In an enzymatic primer extension reaction, this primer will be elongated. During that process the allele-specific analytical products are generated. The products differ by mass according to the incorporated bases. Primers were designed: *ACGTTGGATG*CTGTGAGCACCAATTTGGAC (PHEX-ex21_PCR1) and *ACGTTGGATG*TTCTCTTCTAGGTGAGGTGC (PHEX-ex21_PCR2), with the tag-sequences in italics. For the iPLEX-reaction the primer sequences were: ACAGACCAGAAGCTGCC (left) and CCAATTTGGACTTGTTCTC (right). The sample carrier was introduced into the mass spectrometer (MassARRAY Analyzer Compact, Agena Bioscience) and data are fully automatically acquired and analyzed in a real-time setting and revised using Typer software (Agena Bioscience).

## Results

The technical data of the established NGS panel for the molecular confirmation of the diagnosis of XLH are shown in Table 2. Supplementary Sanger sequencing has been established to completely cover sequences with homopolymers in particular. All in all, close to 100% coverage of all amplicons of the 11 genes in the NGS panel was achieved.

The method proved to be sufficiently robust to process 20 patients in parallel in one reaction approach without loss of quality.

In DNA samples from 49 patients (patients No. 1–50, except for patient 17) with confirmed XLH, the NGS assay correctly re-identified the *PHEX* mutations that were already known and classified as pathogenic. Using the NGS method, 15 nonsense, 12 missense, 4 splice, 7 deletions and 4 insertions were found (Supplementary Table 4). For validation, the sequence variants were again confirmed by Sanger sequencing. In one patient, no obvious mutation could be detected by the gene panel. This sample was subsequently also analyzed by MLPA and revealed a duplication of *PHEX* exon 12, confirming previous results (Supplementary Fig. 1).

DNA samples from three patients that were classified as XLH patients, but in whom no pathogenic mutation in *PHEX* could be detected beforehand, were also examined with the NGS method (P1-P3; Table 3). NGS analysis of the *PHEX* gene of the first sample strongly indicated a mosaicism at cDNA position 2104 (P1; Table 3). Sixty of 706 reads displayed a T allele at position c.2104 while 646 reads displayed the C allele of the reference sequence (c.2104C>T, C: 91.5%, T: 8.5%). This result suggests a mosaic mutation. To confirm the mosaic status in the blood DNA, we verified the NGS analysis employing an assay with iPLEX technology (hME) (Supplementary Fig. 2).

**Table 3:** Variants found in patients 1–3 without proven *PHEX*-mutation.

Gene	Exon	Variant	Variant type	P1, ♀	P2, ♂	P3, ♀
*PHEX*	21	c.2104C=/<T; p.(Arg702*)	Nonsense	Mosaic		
*DMP1*						
	2	c.31delT; p.(Trp11Glyfs*9)	Frameshift		hom	
	6	c.205A>T; p.(Ser69Cys)	Missense			het
	6	c.475C>A; p.(Gln159Lys)	Missense			het
*ENPP1*						
	23	c.2320C>T (p.Arg774Cys)	Missense			het
	25	c.2662C>T (p.Arg888Trp)	Missense			Compound het
	25	c.2663G>A (p.Arg888Gln)	Missense			Compound het
*FAM20C*						
	10	c.1672C>T (p.Arg558Trp)	Missense			het
	10	c.1690A>G (p.Asn564Asp)	Missense			het
*SLC34A3*	13	c.1538A>T; p.(Glu513Val)	Missense			het

het, heterozygous; hom, homozygous.

In the last two samples no variation was detected in the *PHEX* sequence, nor was MLPA suspicious. Therefore, the sequence data of the other genes of the NGS panel were analyzed. The already known nonsense mutation c.31delT (p.Trp11Glyfs*9) in the *DMP1* gene was discovered in a homozygous fashion in the first sample (P2; Table 3). In the second sample, two heterozygous missense mutations, c.475C>A (p.Gln159Lys) and c.205A>T (p.Ser69Cys), were discovered in the *DMP1* gene. In addition, in this sample three variants were detected in the *ENPP1* gene: c.2320C>T (p.Arg774Cys), and the compound heterozygous mutations c.2662C>T (p.Arg888Trp) and c.2663G>A (p.Arg888Gln), both affecting codon position 888 (P3; Table 3).

## Discussion

Recently, first international clinical practice recommendations for the diagnosis and management of XLH have been published, recommending that XLH should be diagnosed not only on the basis of clinical signs of rickets and/or osteomalacia in association with renal phosphate wasting, but also on the basis of molecular analysis, confirming the clinical diagnosis on a genetic level (2). Improvements in gene sequencing technologies in combination with rapidly declining costs have led to the development of a large amount of targeted NGS panels. These panels allow investigating multiple known disease-causing genes in one assay. Therefore, in this study, we developed an NGS panel for the diagnosis of XLH and related disorders. We validated the panel for *PHEX* using 50 DNA-samples with a known *PHEX* mutation. Since the NGS tool revealed a 100% agreement in 49 patient samples, the coverage and the sensitivity must be rated very high, proving that we created an easy, fast, and reliable diagnostic tool for the diagnosis of XLH. An exception is large deletions in 46,XX patients or duplications encompassing one or more exons. In these cases, an additional *PHEX* MLPA analysis must be performed if a mutation cannot be detected with NGS. What sets our XLH gene panel apart from commercial panels is the validation with 50 previously sequenced DNA samples from clinically confirmed XLH patients. However, the validation could only be carried out for the *PHEX* gene. There is probably no comparable cohort with already known mutations in the other genes of the panel.Furthermore, due to the high coverage of the genes in the panel we were able to identify a mosaic mutation in the *PHEX* gene (c.2104C=/<T; p.Arg702*) in one patient sample that had not been detected by Sanger sequencing previously. The reason is most likely that in this case the frequency of the mutated gene copies in DNA from leukocytes was very low (reference base C about 91.5%, but mutated base T in only 8.5% of all reads), but the mosaic mutation has been confirmed by iPLEX and MALDI-TOF MS (Supplementary Fig. 2). Since mosaic mutations are difficult to detect by Sanger sequencing, their description in *PHEX* is rare in the literature (22, 23, 24). Therefore, the identification of this mosaic mutation demonstrated the high sensitivity of the developed NGS panel.

However, the advantage of using the panel is not only the molecular diagnosis of XLH, but also of related disorders of renal phosphate wasting in one investigation. For this goal, we included ten other candidate genes in the panel. These genes were decided to be included into our tool, since all these genes are encoding proteins, which are involved in renal phosphate reabsorption and most of them are known to cause a type of renal phosphate wasting disorder in case of a mutation in one of these genes (for details see Table 1). A validation of variations in these genes except for *PHEX* was not possible because of the rarity of these conditions and the unavailability of samples with known mutations.

In one patient, we proved a known homozygous *DMP1* mutation, which has been detected before by Sanger sequencing. *DMP1* encodes for dentin matrix protein type 1 and is produced by osteoblasts and osteocytes, regulating cell attachment and cell differentiation (25). Homozygous *DMP1*-mutations are the cause for ARHR type 1 (ARHR1) (9, 10, 26); a rare autosomal recessive disorder with biochemical and skeletal signs similar to those observed in XLH. Although there are similarities in the pathophysiology between XLH and AHRH1 especially with elevated or inappropriate normal FGF23-levels, the patient can be treated only by the conventional therapy since Burosumab is exclusively licenced for XLH.

In the third sample without proven *PHEX* mutation, we detected several molecular genetic changes, which could be responsible for the phenotype of the patient. While the *DMP1* variants may be common variants, the most probable reason for the phenotype is the compound heterozygous mutations aforementioned in the *ENPP1* gene, affecting different positions of the same amino acid codon. Both variants are very rare (allele frequency: <0.00003 at gnomAD) (27) and are considered as probably damaging by Polyphen 2 (28) and deleterious by SIFT (sorting intolerant from tolerant) (29). Homozygous or compound heterozygous *ENPP1* mutations have been previously described to lead not only to generalized arterial calcification of infancy (GACI1) (30, 31, 32), but also to autosomal-recessive hypophosphatemic rickets type 2 (ARHR2) in rare cases (8). Although in ARHR2 patients inappropriate high FGF23 levels are seen (8), patients should not be treated with Burosumab, because it is a putative enhancement of the development of vascular calcifications.

Limitations of our approach may be the overall effort that is needed to investigate the genes involved in HR. However, this is reduced if aside from *PHEX* other genes are included in the NGS analysis. Furthermore, several samples can be studied in parallel, leading to an economic advantage. Moreover, while the overall coverage is quite high, a customized panel can only detect variations in the genes included. Hitherto unknown genes involved in HR will not be investigated. Samples, where no variations are detected with the panel, have to be subjected to other NGS methods with an untargeted approach, namely whole-exome sequencing or even whole-genome sequencing (WGS). These methods have the disadvantage of requiring another, often much more extensive bioinformatics approach for analysis and may be used as a second line. Nevertheless, WGS also offers the possibility to detect structural variations such as inversions (not detectable by MLPA) or deletions by the identification of split-pair reads (33). And lastly, all molecular methods applied will be profitable only in light of very informative patients with respect to clinical and biochemical phenotyping.

In conclusion, the use of NGS technology has major advantages for exact diagnosis of the different forms of HR. In contrast to commercially available NGS panels, the panel was validated with known mutated samples and therefore the application of the panel developed in this study seems to be a sensitive and specific tool which can not only detect mutations in *PHEX*, but also in other genes associated with HR. This differentiation is favourable for the patients as it readily leads to very specific treatment options.

## Supplementary Material

Supplementary Table 1: Amplicons of the HR-Panel. Chromosomal positions are described according to GRCh37/hg19

Supplementary Table 2: Primer sequences used to close panel gaps.

Supplementary Table 3: Primer sequences used for Sanger sequencing of homopolymer stretches. Within KL, one amplicon detects two homopolymer stretches.

Supplementary Table 4: Mutations in 50 samples with proven PHEX mutations, validated by the NGS-Panel. A) Individual patient samples and mutations in the different exons of PHEX. The table also lists, which mutations were hemizygous and which were heterozygous. B) Polymorphisms in PHEX, which were detected in the patients. These were homozygous, hemizygous or heterozygous.

Supplementary Figure 1: Normalized intensity of MLPA analysis of PHEX in patient sample 17. The green arrows point to the probes Exon 12-1 and -2, which were at 150 and 132% of normal. As patient 17 was female, a 46,XX karyotype with will have usually two copies of PHEX. In this sample, the higher amplification rate points to a duplication on one allele.

Supplementary Figure 2: Detection and validation of the mosaic mutation. A) Section of aligned single reads of the NGS platform at single nucleotide variation c.[=/2104C >T]. B) Details of the used reference gene, exon position and number of aligned reads displaying the alternate or reference allele as well as a pseudo electropherogram displayed by SeqNext.

## Declaration of interest

O H and S T received honoraria from Kyowa Kirin. The other authors have nothing to disclose.

## Funding

This work was in supported by a grant obtained from Kyowa Kirin.

## Authors contribution statement

S Thiele, R Werner, A Stubble, O Hiort and W Hoeppner contributed equally to this research.
