# Supplementary figures and images for: Validation of a next-generation sequencing (NGS) panel to improve the diagnosis of X-linked hypophosphataemia (XLH) and other genetic disorders of renal phosphate wasting
Source: Eur J Endocrinol. 2020 Aug 14;183(5):497–504. doi: 10.1530/EJE-20-0275 (PMC7592643; doi:10.1530/EJE-20-0275)

Supplementary Figure 1

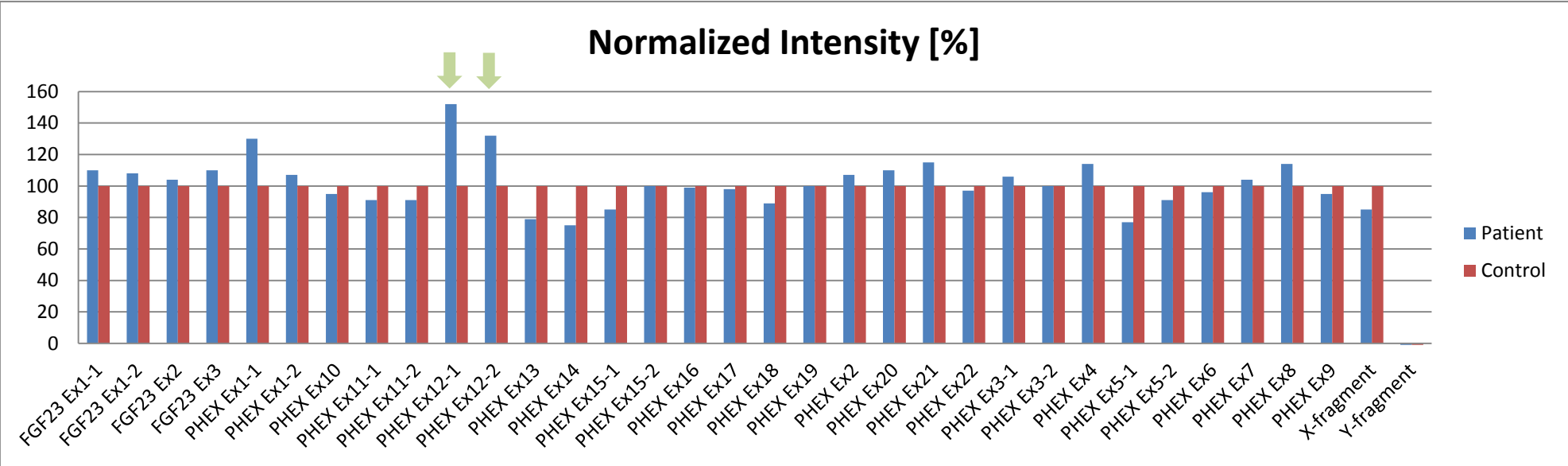

Supplement: Supplementary Figure 1: Normalized intensity of MLPA analysis of PHEX in patient sample 17. The green arrows point to the probes Exon 12-1 and -2, which were at 150 and 132% of normal. As patient 17 was female, a 46,XX karyotype with will have usually two copies of PHEX. In this sample, the higher a [file supplementary_figure_1.pdf]
