# Supplementary material for: Validation of a next-generation sequencing (NGS) panel to improve the diagnosis of X-linked hypophosphataemia (XLH) and other genetic disorders of renal phosphate wasting
Source: Eur J Endocrinol. 2020 Aug 14;183(5):497–504. doi: 10.1530/EJE-20-0275 (PMC7592643; doi:10.1530/EJE-20-0275)

Supplementary Figure 2

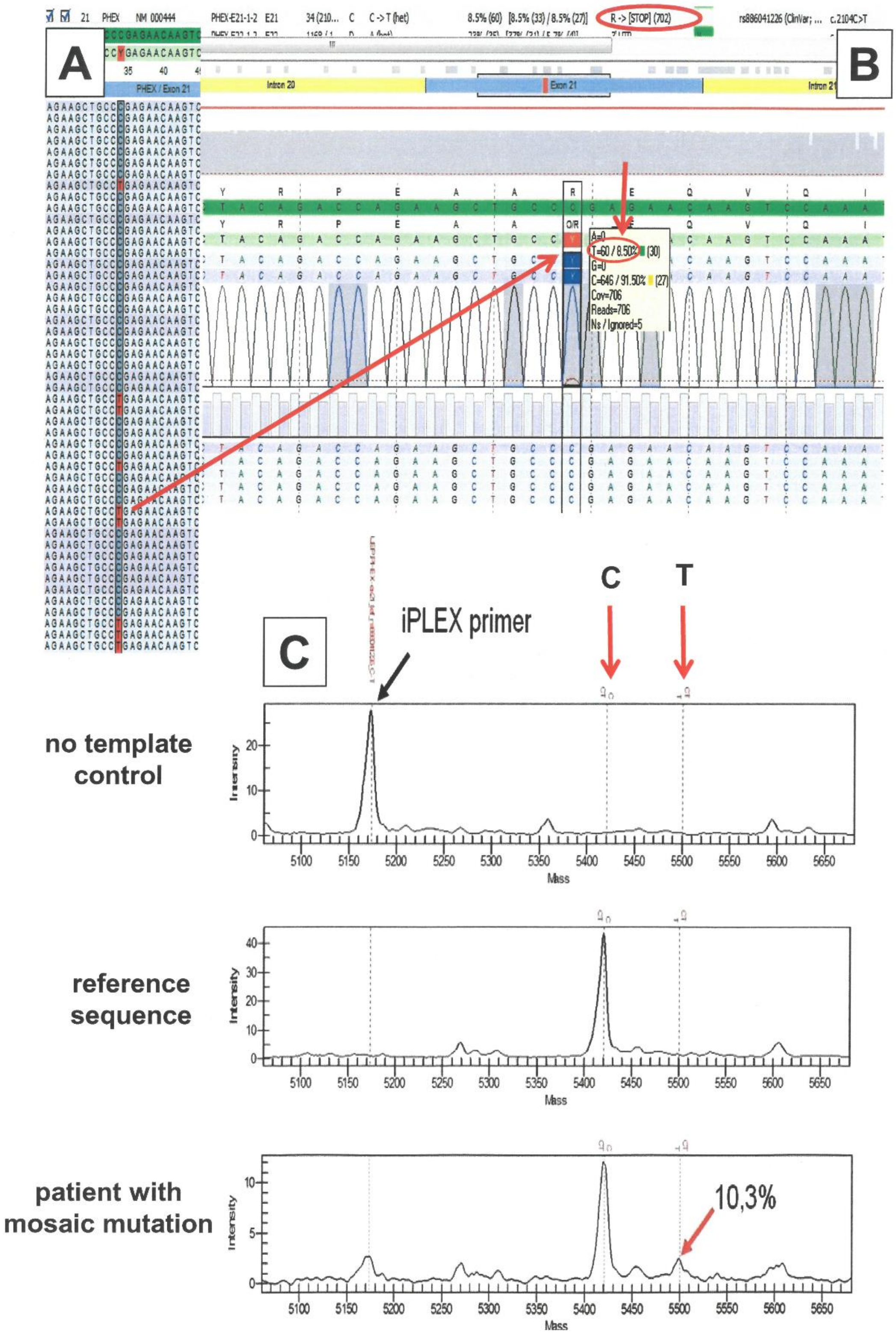

Supplement: Supplementary Figure 2: Detection and validation of the mosaic mutation. A) Section of aligned single reads of the NGS platform at single nucleotide variation c.[=/2104C >T]. B) Details of the used reference gene, exon position and number of aligned reads displaying the alternate or reference allele [file supplementary_figure_2.pdf]
